# Evaluating the Usefulness of YouTube as a Source of Patient Information for Neurosurgical Care in Africa: A Study Protocol

**DOI:** 10.29337/ijsp.168

**Published:** 2021-11-11

**Authors:** Chibuikem A. Ikwuegbuenyi, Lorraine Arabang Sebopelo, Michael A. Bamimore, Oloruntoba Ogunfolaji, Arsene Daniel Nyalundja, Gideon Adegboyega, Daniel Safari Nteranya, Alice Umutoni, Placide Ngoma, Ulrick Sidney Kanmounye

**Affiliations:** 1 Research Department, Association of Future African Neurosurgeons, Yaounde, Cameroon; 2 Faculty of Medicine, University of Botswana, Gaborone, Botswana; 3 School of Medicine, Philadelphia College of Osteopathic Medicine, Philadelphia, PA, United States; 4 College of Medicine, University of Ibadan, Ibadan, Nigeria; 5 Queen Mary University of London, Barts and The London School of Medicine London, United Kingdom; 6 Department of Surgery, University Clinics of Bukavu, Official University of Bukavu, Bukavu, DRC- Forensic Center, Official University of Bukavu, Bukavu, Democratic Republic of Congo; 7 University of Rwanda, college of medicine and health sciences, kigali-Rwanda; 8 Medicine and Surgery, Kabwe Central Hospital, Zambia

**Keywords:** Africa, Health attitudes, Neurosurgery, Online video content, Social media, YouTube

## Abstract

**Background:**

A significant proportion of the public rely on the internet for their health information, and social media has emerged as the principal information source. YouTube is the world’s largest and most popular video library, and it has emerged as a primary health information source because it offers animated and interactive content. However, little is known of its usefulness of neurosurgery videos to African YouTube users. We aim with this study to evaluate the usefulness of YouTube as a source of patient information for neurosurgical care in Africa.

**Methodology:**

This observational study will be conducted using YouTube. A search will be carried out to identify neurosurgery videos suggested to African YouTube viewers from inception to September 2021. An internet browser (Google Chrome, Google Inc., CA, USA) with its cache cleared will be used to execute the search. The default YouTube search setting of “relevance” will be used to replicate what a search attempt performed by a patient would be. The first 50 results from each keyword search will be registered in a Microsoft Excel spreadsheet (Microsoft, WA, USA). The primary outcome measure is the reliability of the videos. Data will be analyzed using SPSS version 26 (IBM, WA, USA). Odds ratios and their 95% confidence intervals will be calculated. The statistically significant level will be set at 0.05. Also, a linear regression analysis will be performed to examine the effects of independent variables on continuous dependent variables.

**Dissemination:**

The study findings will be published in an academic peer-reviewed journal, and the abstract will be presented at an international conference. English and French visual and video abstracts of the methods and key findings will be designed and disseminated widely on social media.

**Highlights:**

- A significant proportion of the public rely on the internet and social media for health information.
- YouTube has emerged as the world’s largest video library, and has emerged as a primary health information source.
- There are few safeguards to avoid dissemination of false or biased information on the platform this could negatively influence health seeking behavior
- We aim to evaluate the usefulness of YouTube as a source of patient information for neurosurgical care in Africa.
- The findings of this study will help evaluate the volume and quantity of African neurosurgical video content and identify best practices.

## 1. Background and Rationale

A significant proportion of the public rely on the internet for their health information, and social media has emerged as the principal information source [1]. Social media platforms offer free instantaneous communication with acquaintances and their sharing features have facilitated knowledge dissemination. Social media is not without disadvantages. First, users need reliable internet access to partake in social media activities so internet access has become a determinant of health information equity. In most high-income countries (HICs) the internet is widely accessible and affordable [2]. However, internet coverage is limited and broadband costs are very high in low- and middle-income countries especially within Africa where less than 22% of Africans have reliable and affordable internet access [2]. Secondly, the instantaneous nature and wide audience of social media promote content generation. The content volume can be overwhelming for users making it difficult to identify quality and verifiable content because social network algorithm suggestions prioritize popularity over quality. The high volume of content is also a challenge for creators because they must figure out innovative ways for their content to stand out. One way most content creators do this is through animation and visualization – image or video media.

YouTube is the world’s largest and most popular video library. The platform is projected to have more than 3 billion users by 2025 [3]. However, its penetration rate in Africa remains low at less than 60% primarily due to videos using up more bandwidth than other forms of content [4]. The platform has emerged as a primary health information source because it offers animated and interactive content [5]. Unfortunately, there are few safeguards or standards to avoid the dissemination of false or biased information on the platform, and the instantaneous nature of social media means that once false information is identified it is almost impossible to undo the harm that has been done. As a result, the onus of validating health-related information falls on users who are often unequipped or lack the time to vet these videos [6].

Videos published by non-medical professionals and those communicating false medical information have higher social media reach (views, likes, comments, and shares) than videos made by professionals [7]. Beyond its wide dissemination, this unverified information influences health-seeking behavior adversely. In the same vein, factual information helps improve health-seeking behavior [7] but medical professionals often lack the skills to generate engaging content [8].

Previous studies have analyzed the reliability of online patient educational videos in various aspects of neurosurgery [9,10,11]. However, none have evaluated videos available to African YouTube viewers. With that in mind, we aim to evaluate the usefulness of YouTube as a source of patient information for neurosurgical care in Africa. The study findings will help evaluate the volume and quantity of African neurosurgical video content and identify best practices so African neurosurgeons can improve video content.

### 1.1 Aims and Objectives

To evaluate the usefulness of YouTube videos on neurosurgery available to African YouTube viewers.

## 2. Methods and Analysis

### 2.1. Study design

This observational study will be conducted using YouTube. A search strategy using the terms in Appendix 1 will be used to identify neurosurgery videos suggested to African YouTube viewers from inception to September 2021. The video search strategy and evaluation will be piloted among an arbitrary sample of 2 videos to identify technical issues with the strategy and evaluation.

### 2.2. Inclusion and Exclusion Criteria

The inclusion criteria were adapted and modified from previous studies [12,13]. Videos made in English, French, Arabic, Swahili and Hausa will be included. This language restriction is motivated by the authors’ fluency in these languages. These languages are also the most spoken in Africa. All videos unrelated to neurosurgery will be excluded.

### 2.3. Video Search Strategy

The video search strategy will be adapted and modified from previous studies evaluating healthcare-related YouTube content [14,15,16]. That is; an internet browser (Google Chrome, Google Inc, CA, USA) with its cache cleared will be used to execute the search using the keywords (Appendix 1). This search would be done on a signed in account with the browser and YouTube in incognito mode. This mode allows users to make searches that are not influenced by previous searches [17,18]. The default YouTube search setting of “relevance” will be used to replicate what a search attempt performed by a patient would be. As most of the authors live in various African countries, the search will be done on various computers simultaneously or from a single computer with the location changed with each keyword. The first 50 results from each keyword search will be registered in a Microsoft Excel spreadsheet (Microsoft, WA, USA). The spreadsheet will be reviewed to identify duplicates and these duplicates will be deleted. Next, each video will be watched independently by two reviewers and evaluated using standardized tools (see outcome measures). The content evaluation scores will be averaged between the two independent reviewers. Conflicts with regards to video eligibility and evaluation will be discussed by the two reviewers and if they cannot agree a third reviewer (the senior author) will arbitrate.

### 2.4. Data Extraction

Basic descriptive data will be extracted from the videos, including the number of views, likes, comments, date of upload, and duration.

### 2.5. Outcome Measures

The primary outcome measure is the reliability of the videos. This will be determined using checklists: (1) modified DISCERN criteria [19], (2) the Journal of the American Medical Association (JAMA) benchmark criteria score [20]. The modified DISCERN score will be used to evaluate the clarity of videos, reliability, bias, reference supplementation, and uncertainty of content [21]. The maximum score is 5 points representing the sum of points assigned to each criterion, with higher points indicating higher reliability. The DISCERN criterion is a brief questionnaire that has been used to assess the reliability and quality of written information [6,7], ***Table 1***. The JAMA benchmark criteria score evaluates authorship, attribution, disclosure, and currency, with a maximum of 4 points [22,23], ***Table 2***. Both tools have been used in the evaluation of YouTube videos [7,23].

**Table 1:** Modified DISCERN criteria.


1	Are the aims clear and achieved?

2	Are reliable sources of information used?

3	Is the information presented balanced and unbiased?

4	Are additional sources of information listed for patient reference?

5	Are areas of uncertainty mentioned?


*Note*: Adapted from Radonjic et al 2019 [7].

**Table 2:** Journal of the American Medical Association Score (JAMAS).


1	Authorship	Authors and contributors, their affiliations, and relevant credentials should be provided

2	Attribution	References and sources for all content should be listed clearly, and all relevant copyright information should be noted

3	Disclosure	Website “ownership” should be prominently and fully disclosed, as should any sponsorship, advertising, underwriting, commercial funding arrangements or support, or potential conflicts of interest

4	Currency	Dates when content was posted and updated should be indicated


*Note*: Adapted from Radonjic et al 2019 [7].

Secondary outcome measures include YouTube video metrics duration, video age (calculated from the upload date to the data extraction date), number of views, likes, dislikes, number of comments, number of subscribers, and references to sources. These metrics will be used to calculate the Video Power Index (VPI). The VPI assesses video popularity and is calculated as ([view ratio × like ratio]/100), where view ratio = views per day, and like ratio = (likes × 100)/(likes + dislikes) [6,7,24,25].

### 2.6. Video Categorization

Videos will be classified into 4 categories based on their source: university/professional organization, medical advertising/for-profit companies, independent users, and others (i.e., news/media, governmental organization).

### 2.7. Data Analysis

Descriptive data will be used to define continuous variables (i.e., mean and standard deviation/95% confidence interval or median and interquartile range) and categorical variables (frequency and percentage). Comparisons of 2 independent and normally distributed continuous variables will be performed with the Student t-test. Comparisons of 2 independent and non-normally distributed continuous variables were performed with the Mann Whitney U test. The Spearman r coefficient will be calculated to evaluate the correlation between 2 non-normally distributed continuous variables. Also, Cronbach’s coefficient will be calculated to determine the consistency between 2 continuous measurements. A linear regression analysis will be performed to examine the effects of independent variables on continuous dependent variables. The statistically significant level will be set at 0.05, and the statistical analysis will be performed using SPSS version 26 (IBM, WA, USA).

## 3. Limitations

We recognize several limitations in this study. First, we are limiting video analysis to YouTube excluding other equally popular video repositories like Facebook, TikTok, and WhatsApp. YouTube lends itself to a more systematic review of video content unlike the other platforms thanks to its advanced search algorithm. YouTube’s search engine algorithm is inspired by the Google search algorithm. Of note, the YouTube algorithm is influenced by the geographic location of the viewer, time of posting, and level of interaction with the video [25,26,27]. Data will be collected on a single day, on the same computer, and, the same browser to minimize heterogeneity. Finally, only the commonest colonial languages prevalent in Africa, i.e, English and French are evaluated, videos made in other commonly spoken languages indigenous to Africa cannot be assessed for the accuracy of information passed to patients.

## 4. Conclusion

YouTube is an important source of health information but little is known of the usefulness of neurosurgery videos to African YouTube users. The authors will use standardized tools to independently assess the reliability of neurosurgery videos suggested to African users. In addition, they will evaluate the popularity of these videos and identify their determinants. The study findings will help improve content generation and neurosurgical patient education in Africa.

## 5. Ethical Considerations

Approval to carry out this study will not be necessary because the videos analyzed in this study will be published online.

## 6. Dissemination

The study findings will be published in an academic peer-reviewed journal, and the abstract will be presented at an international conference. English and French visual and video abstracts of the methods and key findings will be designed and disseminated widely on social media.

## Additional File

The additional file for this article can be found as follows:

10.29337/ijsp.168.s1 Appendix 1. Inclusion and exclusion criteria.

## Abbreviations

HICs, High income countries; VPI, Video power index.
