# Supplementary material for: Evaluating the Usefulness of YouTube as a Source of Patient Information for Neurosurgical Care in Africa: A Study Protocol
Source: Int J Surg Protoc. 2021 Nov 11;25(1):244–9. doi: 10.29337/ijsp.168 (PMC8588890; doi:10.29337/ijsp.168)
Supplement: Appendix 1. — Inclusion and exclusion criteria. [file ijsp-25-1-168-s1.pdf]

Appendix 1

List of search terms

List of Search Terms	
1)	“Neurosurgery in Africa”
2)	“Neurological surgery in Africa”
3)	“ Neurosurgical care in Africa”
4)	“Brain Surgery”
5)	“Traumatic Brain Injury”
6)	“TBI”
7)	“Brain Injury”
8)	“Concussion”
9)	“Head Injury”
10)	“Traumatic Spinal Injury”

11)	“Spinal Cord Injury”
12)	“Hydrocephalus”
13)	“Stroke”
14)	“Brain Infarction”
15)	“Brain Infections”
16)	“Meningitis”
17)	“Peripheral nerve Injuries”
18)	“Epilepsy”
19)	“Seizure”
20)	“Convulsions”
21)	“Brain Tumor”
22)	“Brain Cancer”
23)	“Spinal Cord Tumor”

24)	“Cerebral Aneurysm”
25)	“Cerebral Abscess”
26)	“Neurochirurgie en Afrique”,
27)	“soins neurochirurgicaux en Afrique”
28)	“Traumatisme cranio-encephalique”
29)	“Commotion cérébrale”
30)	“Lésion cérébrale”
31)	“Blessure à la tête”
32)	“Traumatisme du rachis”
33)	“Lésion de la moelle épinière”
34)	“Hydrocéphalie”
35)	“Accident vasculaire cérébral"
36)	“Infarctus cerebral”

37)	“Infection cérébrale”
38)	“méningite”
39)	“Lésion du nerf périphérique”
40)	“Épilepsie”
41)	“Tumeur du cerveau”
42)	“Cancer du cerveau”
43)	“Tumeur de la moelle épinière”
44)	“cancer de la moelle épinière"
45)	“spina bifida”
46)	“malformation du système nerveux”
47)	"anévrisme cérébral",
48)	“abcès cérébral”
49)	“abcès du cerveau ou cérébral"

50)	”جراحة المخ والأعصاب في أفريقيا“
51)	”جراحة الأعصاب في إفريقيا“
52)	”رعاية جراحة الأعصاب في إفريقيا“
53)	"جراحة الدماغ"
54)	"إصابات في الدماغ"
55)	"إصابة الدماغ"
56)	"ارتجاج في المخ"
57)	"إصابة بالرأس"
58)	"إصابات العمود الفقري الرضحية"

59)	"اصابة الحبل الشوكي"
60)	"استسقاء الرأس"
61)	"السكتة الدماغية"
62)	"احتشاء الدماغ"
63)	"التهابات الدماغ"
64)	"التهاب السحايا"
65)	"إصابات الأعصاب الطرفية"
66)	"الصرع"
67)	"انتزاع"

68)	"تشنجات"
69)	"ورم في المخ"
70)	"سرطان الدماغ"
71)	"ورم الحبل الشوكي"
72)	"تمدد الأوعية الدموية الدماغية"
73)	"خراج دماغي"
74)	"Upasuaji wa neva katika Afrika"
75)	"Upasuaji wa neva katika Afrika"
76)	"Huduma ya upasuaji wa neva katika Afrika"

77)	"Upasuaji wa Ubongo"
78)	"Kuumia kwa Ubongo wa Kiwewe"
79)	"Kuumia kwa Ubongo"
80)	"Shindano"
81)	"Kuumia Kichwa"
82)	"Jeraha la Kiwewe la Mgongo"
83)	"Kuumia kwa Mgongo"
84)	"Hydrocephalus"
85)	"Kiharusi"

86)	"Infarction ya ubongo"
87)	"Maambukizi ya Ubongo"
88)	"Homa ya uti wa mgongo"
89)	"Majeraha ya neva ya pembeni"
90)	"Kifafa"
91)	"Kukamata"
92)	"Machafuko"
93)	"Tumor ya Ubongo"
94)	"Saratani ya ubongo"

95)	"Uvimbe wa uti wa mgongo"
96)	"Aneurysm ya ubongo"
97)	"Jipu la ubongo"
98)	"Neurosurgery a Afirka"
99)	"Yin aikin tiyata a Afirka"
100)	"Kulawar Neurosurgical a Afirka"
101)	“Tiyata ta kwakwalwa”
102)	"Raunin Raunin Brain"
103)	"Raunin Brain"

104)	"Rikici"
105)	"Raunin Kai"
106)	"Raunin Spinal Traumatic"
107)	"Cutar sankarau"
108)	"Raunin jijiya na gefe"
109)	“Kashewa”
110)	“Rikici”
111)	"Tumor na Brain"
112)	"Ciwon Kankara"
